# Fluorescence spectroscopy of roGFP2-based redox probes responding to various physiologically relevant oxidant species *in vitro*

**DOI:** 10.1016/j.dib.2017.03.015

**Published:** 2017-03-11

**Authors:** Alexandra Müller, Jannis F. Schneider, Adriana Degrossoli, Nataliya Lupilova, Tobias P. Dick, Lars I. Leichert

**Affiliations:** a Institute of Biochemistry and Pathobiochemistry – Microbial Biochemistry, Ruhr-University Bochum, 44780 Bochum, Germany; b Division of Redox Regulation, DKFZ-ZMBH Alliance, German Cancer Research Center (DKFZ), Im Neuenheimer Feld 280, 69120 Heidelberg, Germany

**Keywords:** Genetically encoded redox probes, Polysulfides, HOCl, Peroxynitrite, glutathione, H_2_O_2_, Nitric oxide

## Abstract

This article contains representative fluorescence excitation spectra of roGFP2-based probes used for ratiometric analysis of redox changes as presented in the article "Systematic *in vitro* assessment of responses of roGFP2-based probes to physiologically relevant oxidant species" [1]. The recombinant probes roGFP2, roGFP2-Orp1, and Grx1-roGFP2 were exposed to various oxidative and nitrosative species, including hydrogen peroxide (H_2_O_2_), aldrithiol-2 (AT-2), glutathione disulfide (GSSG), hypochlorous acid (HOCl), S-nitrosoglutathione (GSNO), peroxynitrite (ONOO^−^), potassium polysulfide (K_2_S_x_), spermine NONOate (SperNO), and diethyl amino NONOate (DeaNO) at different molar ratios. Fluorescence excitation spectra of the probes were recorded in the excitation wavelength range between 350 and 500 nm and for a total of 60 min. Analysis and interpretation of the data is presented in an associated article [1].

**Specifications Table**

**Table:** 

Subject area	*Biology*
More specific subject area	*Redox Biology*
Type of data	*figure, raw data file (.csv)*
How data was acquired	*JASCO FP-8500 fluorescence spectrometer equipped with a Peltier thermo-holder ‘EHC-813’ (JASCO, Darmstadt, Germany)*
Data format	*Raw*
Experimental factors	*Reduction of the probes with DTT*
Experimental features	*Assessment of changes in the fluorescence excitation characteristics of roGFP2-based probes upon oxidant treatment*
Data source location	*Bochum, Germany, Latitude 51.4445974, Longitude 7.258836*
Data accessibility	*Spectral data are displayed in*Figs. 1–10*. Associated raw data can be accessed as .csv text files in the supplementary data section*

**Value of the data**

• The influence of diverse oxidative and nitrosative species on roGFP2-based probes is compared side-by-side.
• Spectral responses of roGFP2, roGFP2-Orp1 and Grx1-roGFP2 are compared side-by-side
• Full spectra are recorded every minute for 60 min.
• The data delineate probe redox responses under strictly controlled *in vitro* conditions.

## 1 Data

**Fig. 1:**
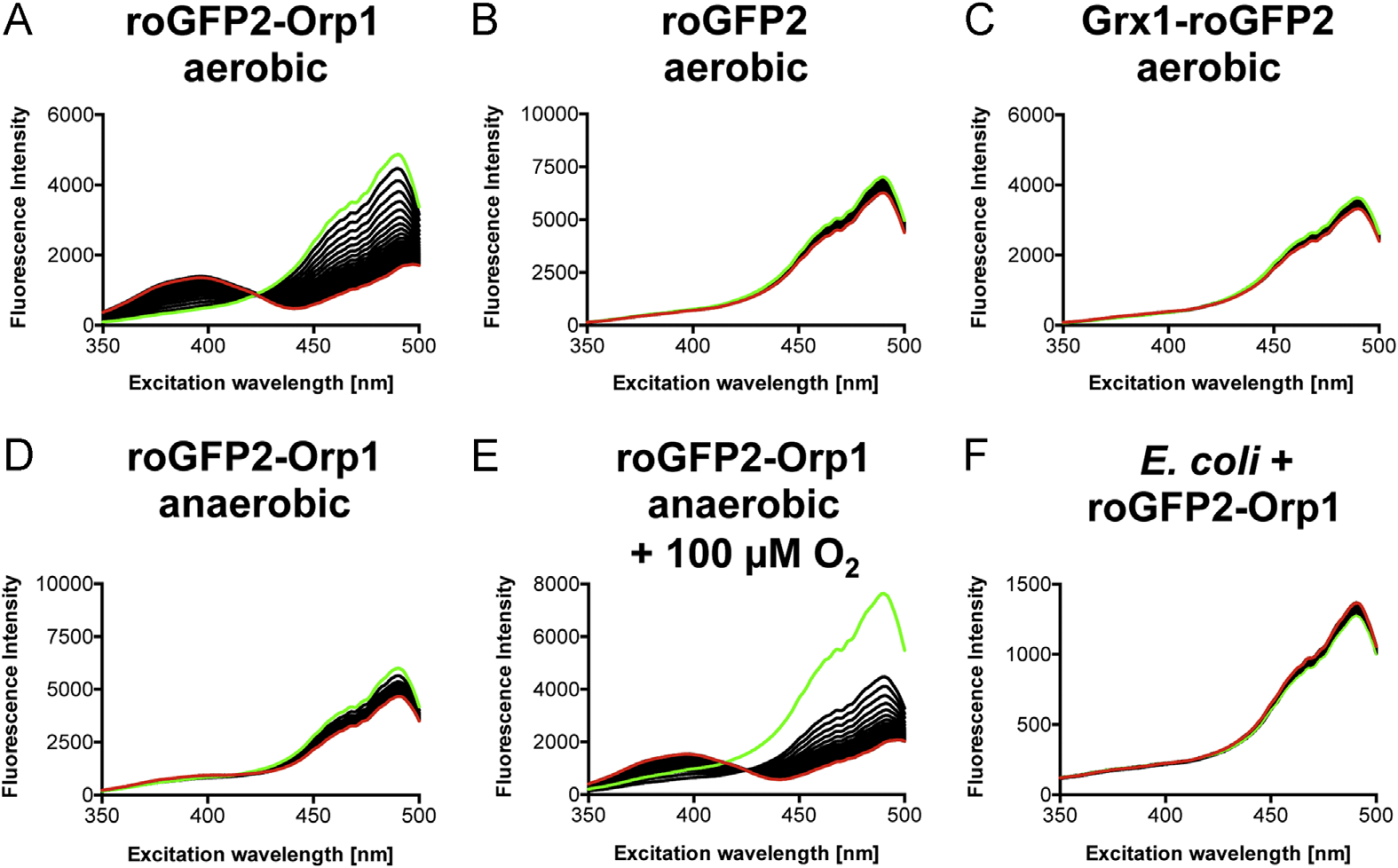
Response of the three probes to air oxygen. Each figure displays the dynamics of 60 measured emission spectra (one spectrum per minute; *E_m_*=510 nm) in the excitation wavelength range of 350–500 nm. First (0 min) and last spectra (60 min) are displayed in green and red, respectively. The probes were added to PBS buffer at a final concentration of 0.2 µM (A–E) or expressed in *E. coli* (F). Measurements were either done under aerobic conditions (A–C) or under fully anaerobic conditions (D). One volume of aerobic buffer corresponding to 100 µM O_2_ was added after measurement of the first spectrum (E).

**Fig. 2:**
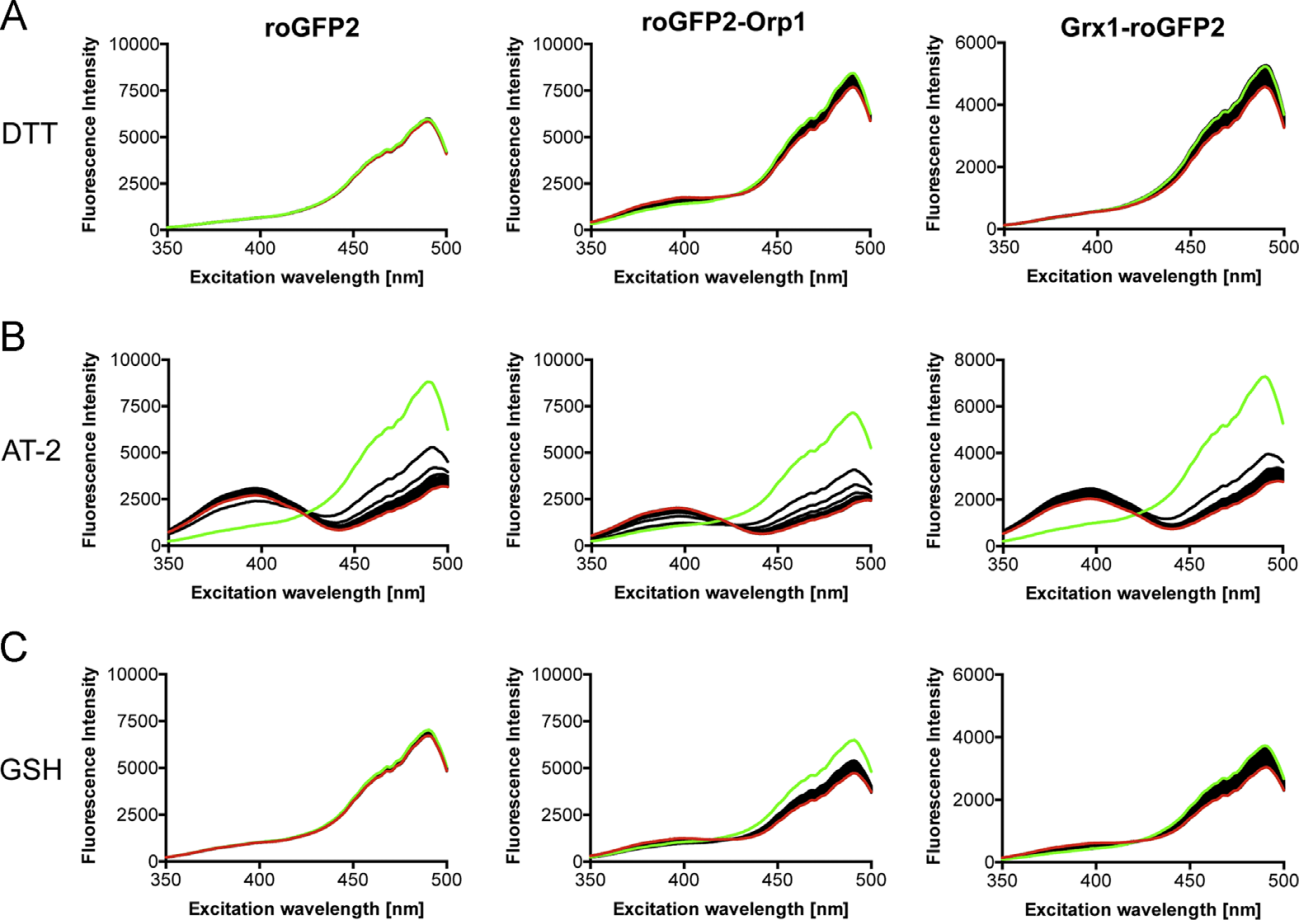
Response of the three probes to DTT, AT-2 and GSH. The probes were added to PBS buffer to a final concentration of 0.2 µM. A single spectrum (green) was recorded prior to addition of DTT (A), AT-2 (B), or GSH (C) to a final concentration of 2 µM. The responses of the probes were measured for a total of 60 min. Each figure displays 60 single spectra with one spectrum per minute. The last recorded spectrum is shown in red.

**Fig. 3:**
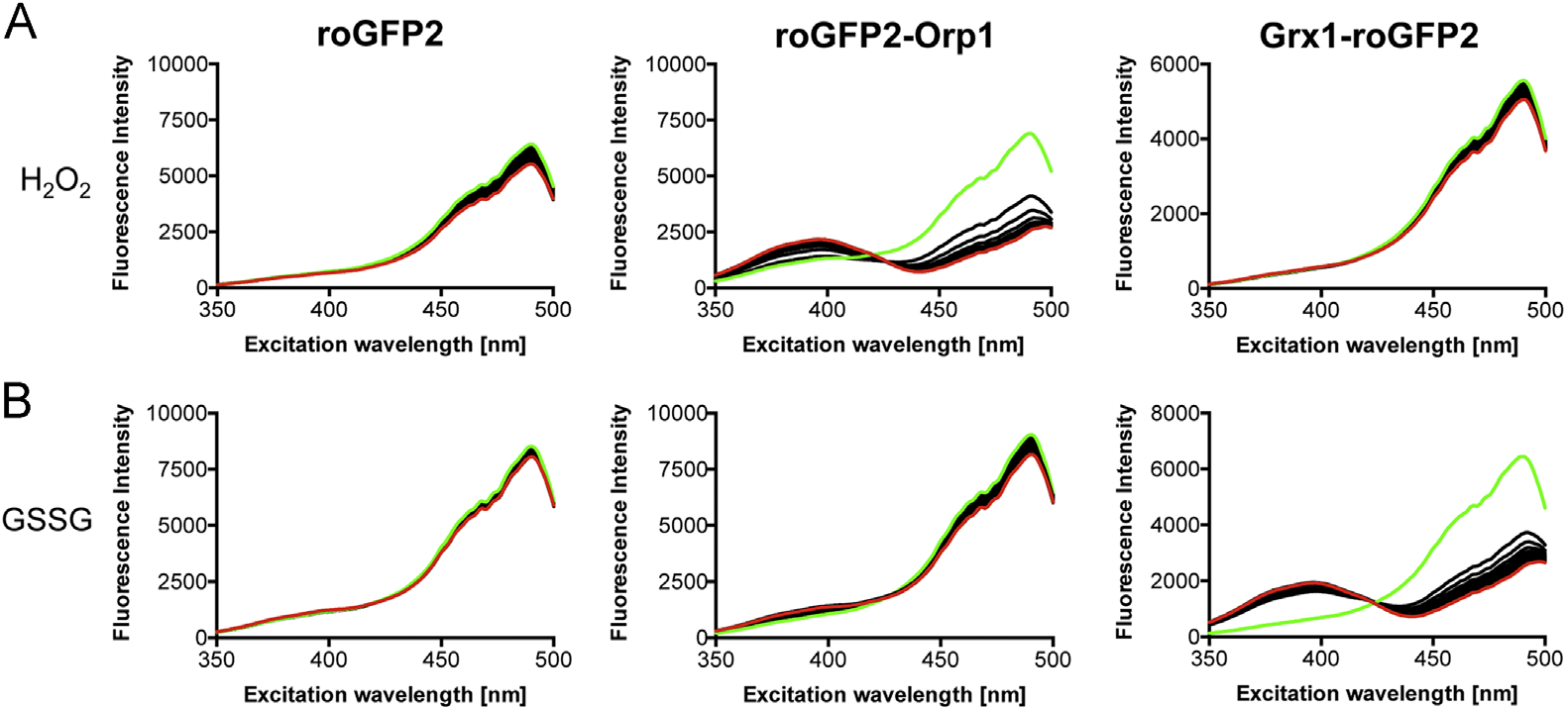
Response of the three probes to H_2_O_2_ and GSSG. A single spectrum (green) of roGFP2, roGFP2-Orp1 and Grx1-roGFP2 at a final concentration of 0.2 µM was measured prior to the addition of 2 µM H_2_O_2_ (A) or 2 µM GSSG (B). Subsequently, another spectrum was recorded every minute for a total of 60 min. The last recorded spectrum is shown in red.

**Fig. 4:**
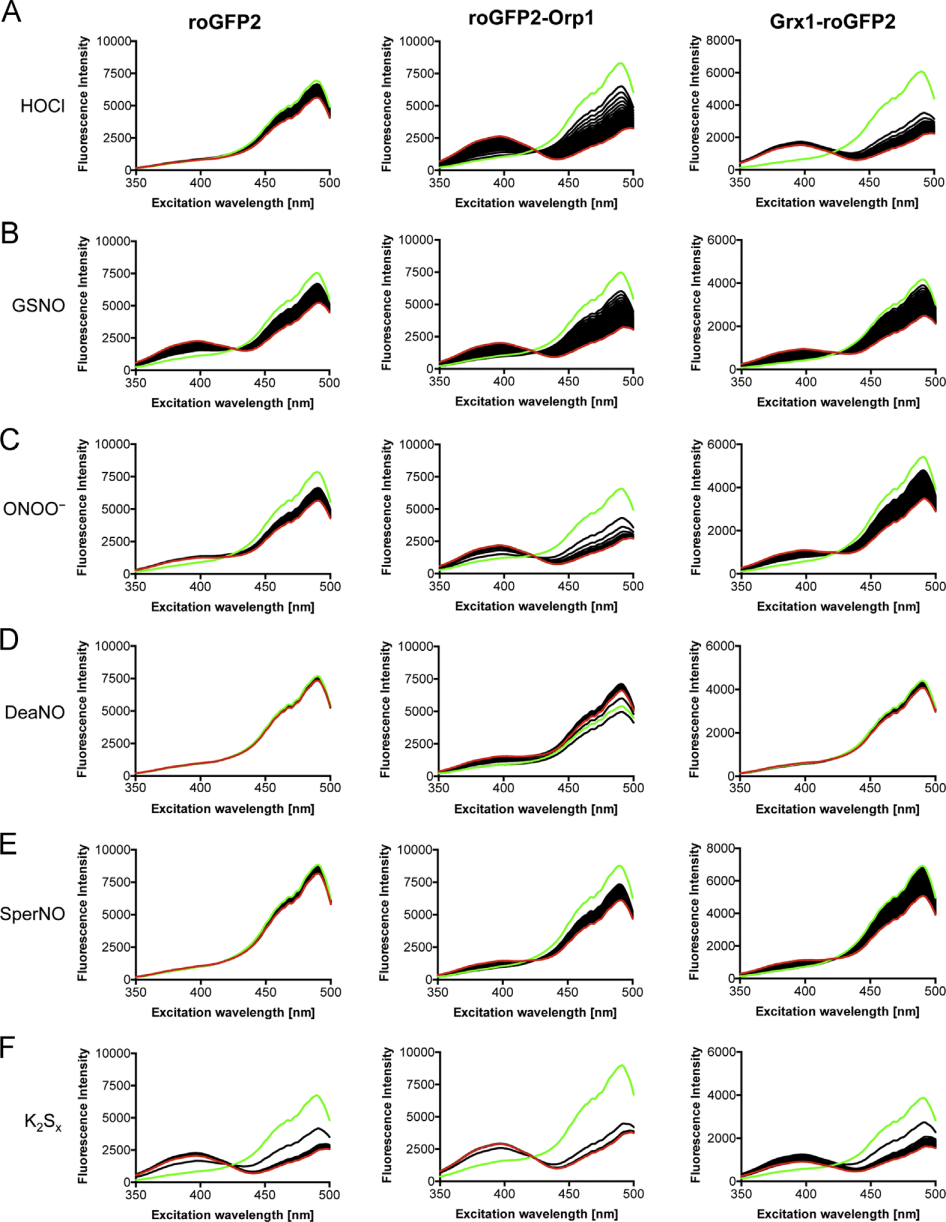
Response of the three probes to various oxidative and nitrosative agents. 0.2 µM of the probes were added to PBS. After the measurement of one spectrum (green), the indicated compounds were added to a final concentration of 2 µM. Subsequently, a single spectrum was measured every minute for a total of 60 min. The last recorded spectrum is shown in red.

**Fig. 5:**
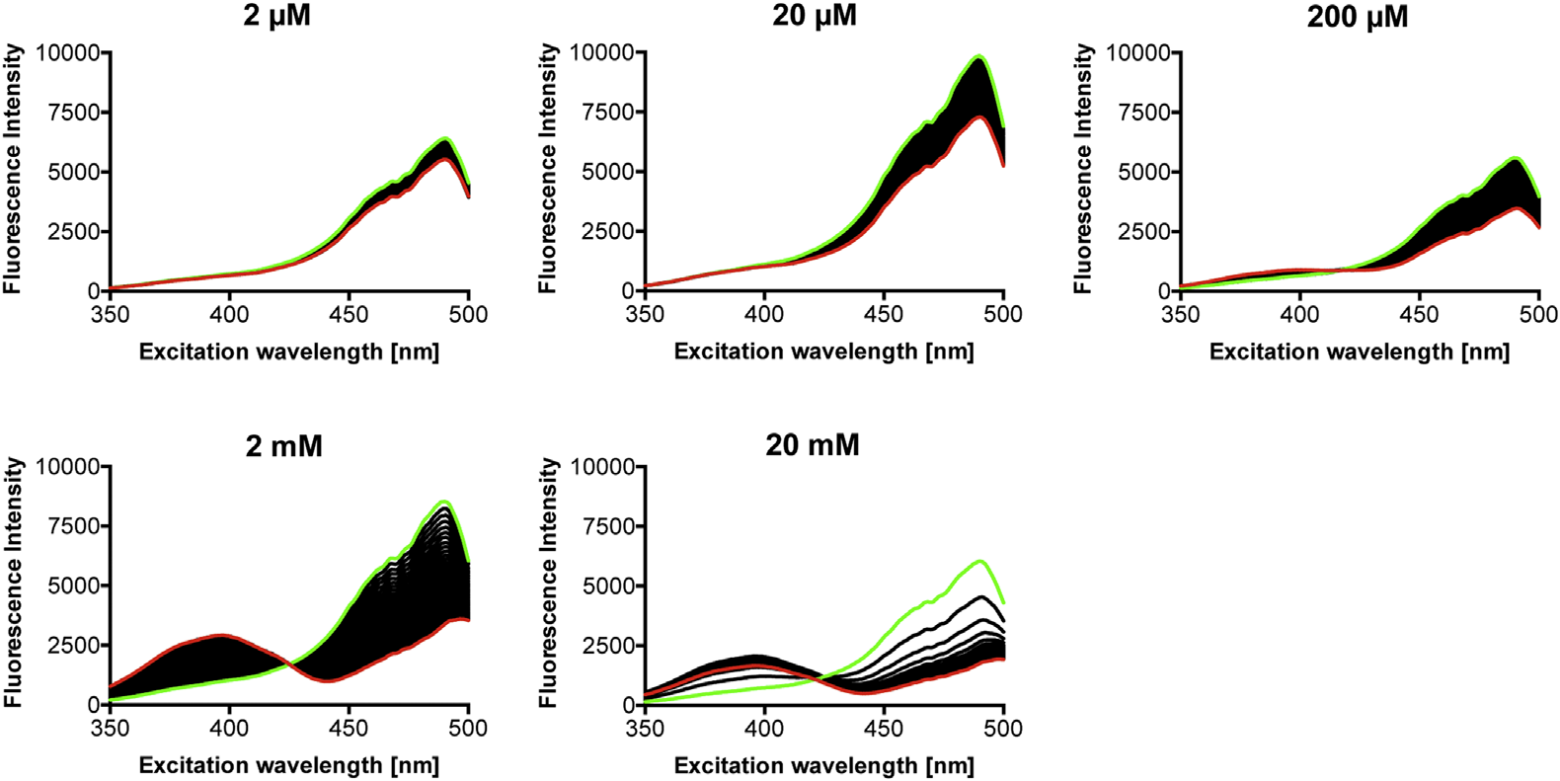
Response of unfused roGFP2 to increasing concentrations of H_2_O_2_. A single spectrum of roGFP2 (0.2 µM) was recorded (green). Oxidation was measured for a total of 60 min after addition of the indicated H_2_O_2_ concentrations (one spectrum each minute). The last recorded spectrum is shown in red.

**Fig. 6:**
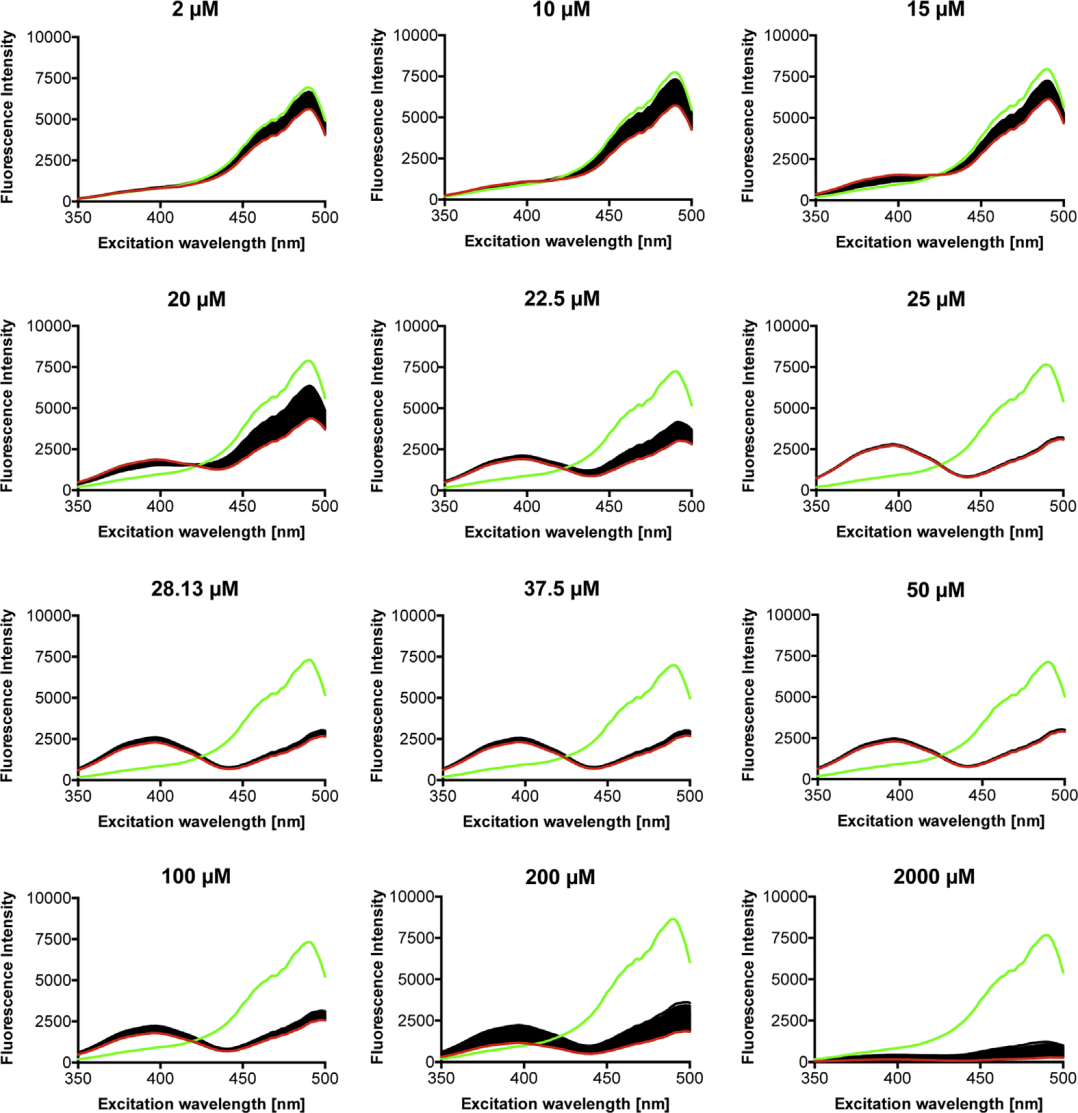
Response of unfused roGFP2 to increasing concentrations of HOCl. HOCl concentrations between 2 µM and 2 mM were applied to roGFP2 after measurement of the first spectrum (green). Subsequently, a total of 60 spectra (one spectrum each minute) were recorded. The last recorded spectrum is shown in red.

**Fig. 7:**
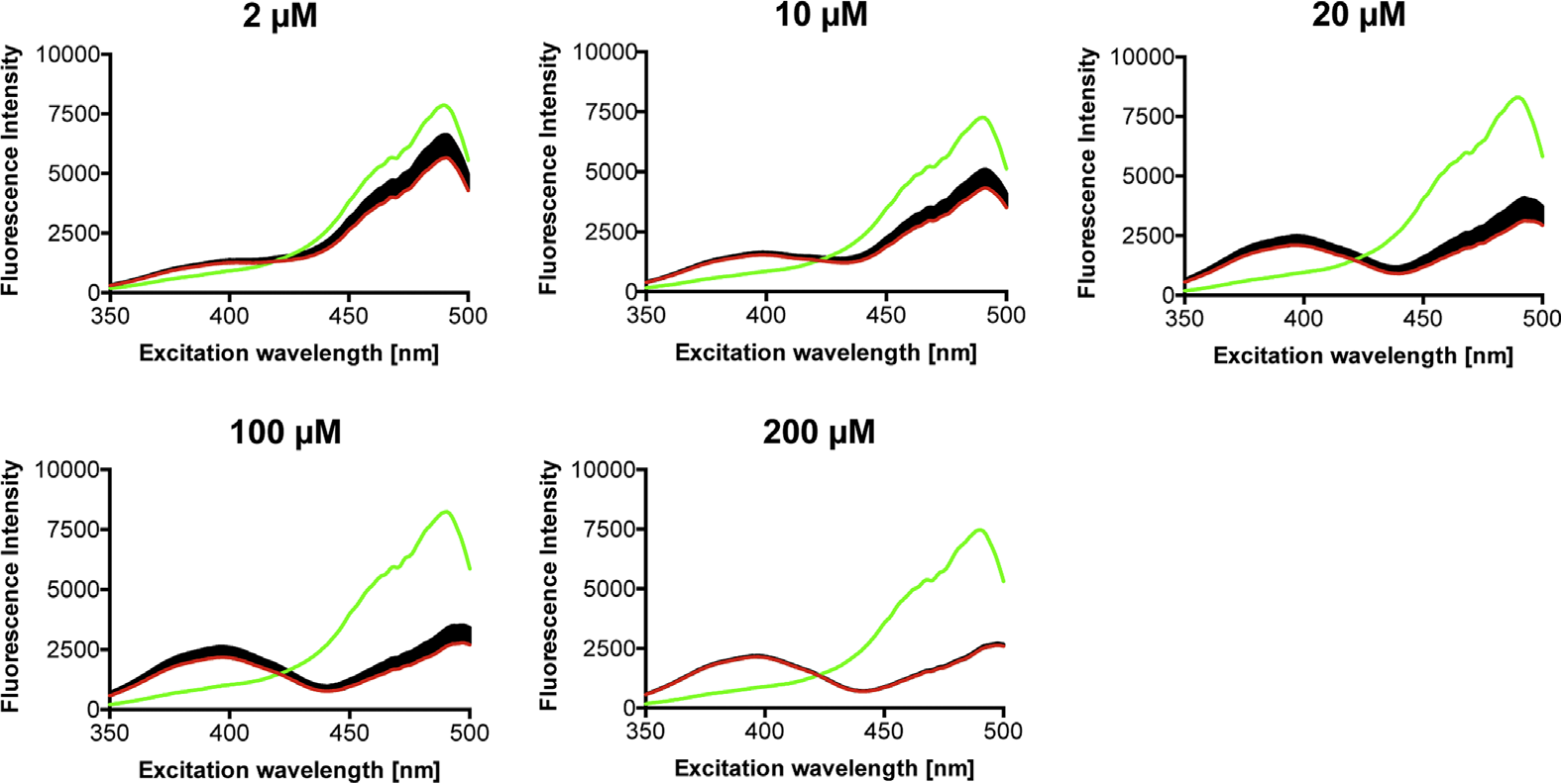
Response of unfused roGFP2 to peroxynitrite. Peroxynitrite concentrations between 2 µM and 200 µM were applied to 0.2 µM roGFP2 after measurement of the first spectrum (green). Subsequently, a total of 60 spectra (one spectrum each minute) were recorded. The last recorded spectrum is shown in red.

**Fig. 8:**
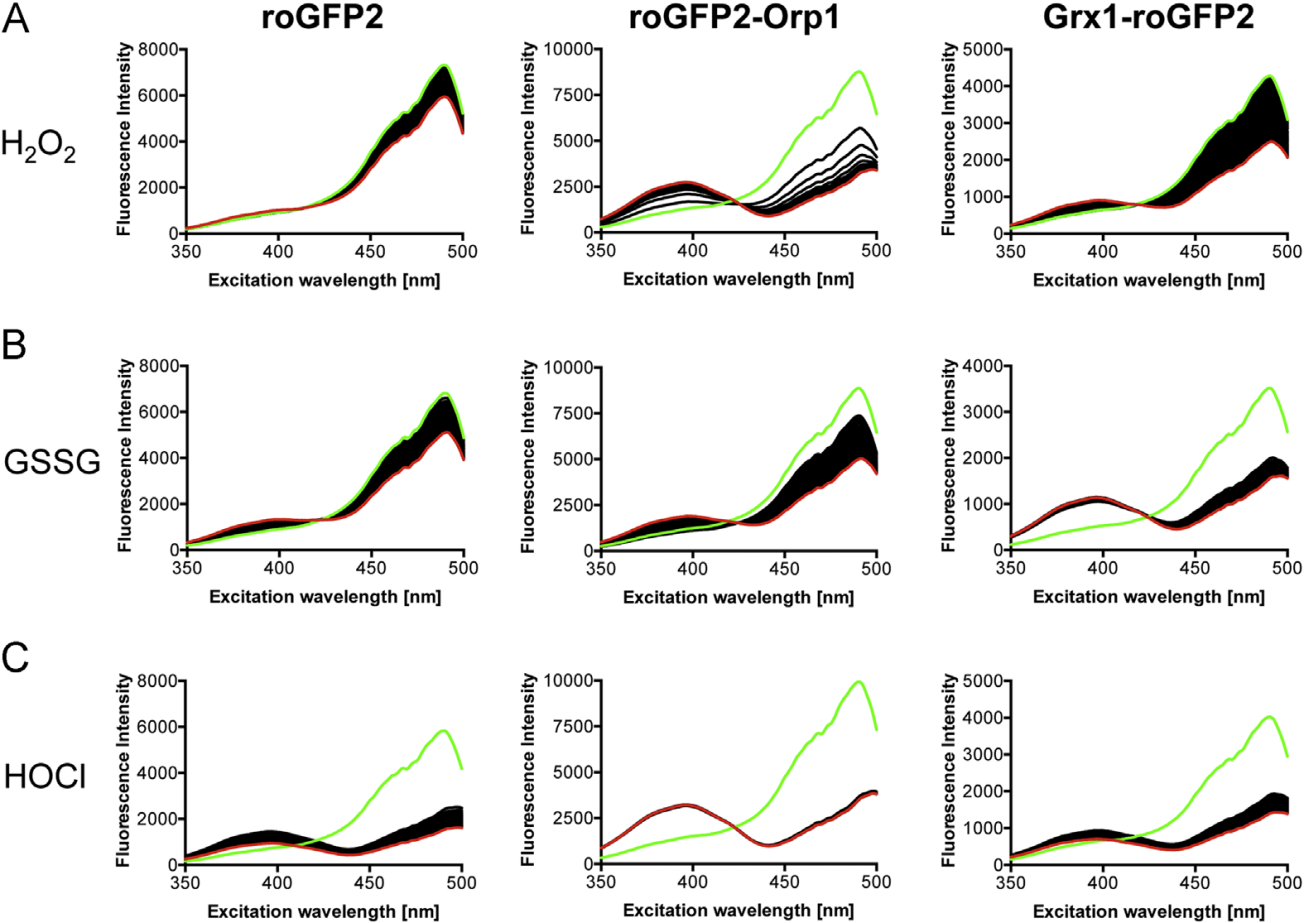
Response of the three probes to a 500-fold molar excess of H_2_O_2_, GSSG and HOCl. The probes were measured at a concentration of 0.2 µM. 100 µM of oxidant were added after measurement of a single spectrum (green). Each figure displays the dynamics of a total of 60 recorded spectra (one spectrum per minute). The last recorded spectrum is shown in red.

**Fig. 9:**
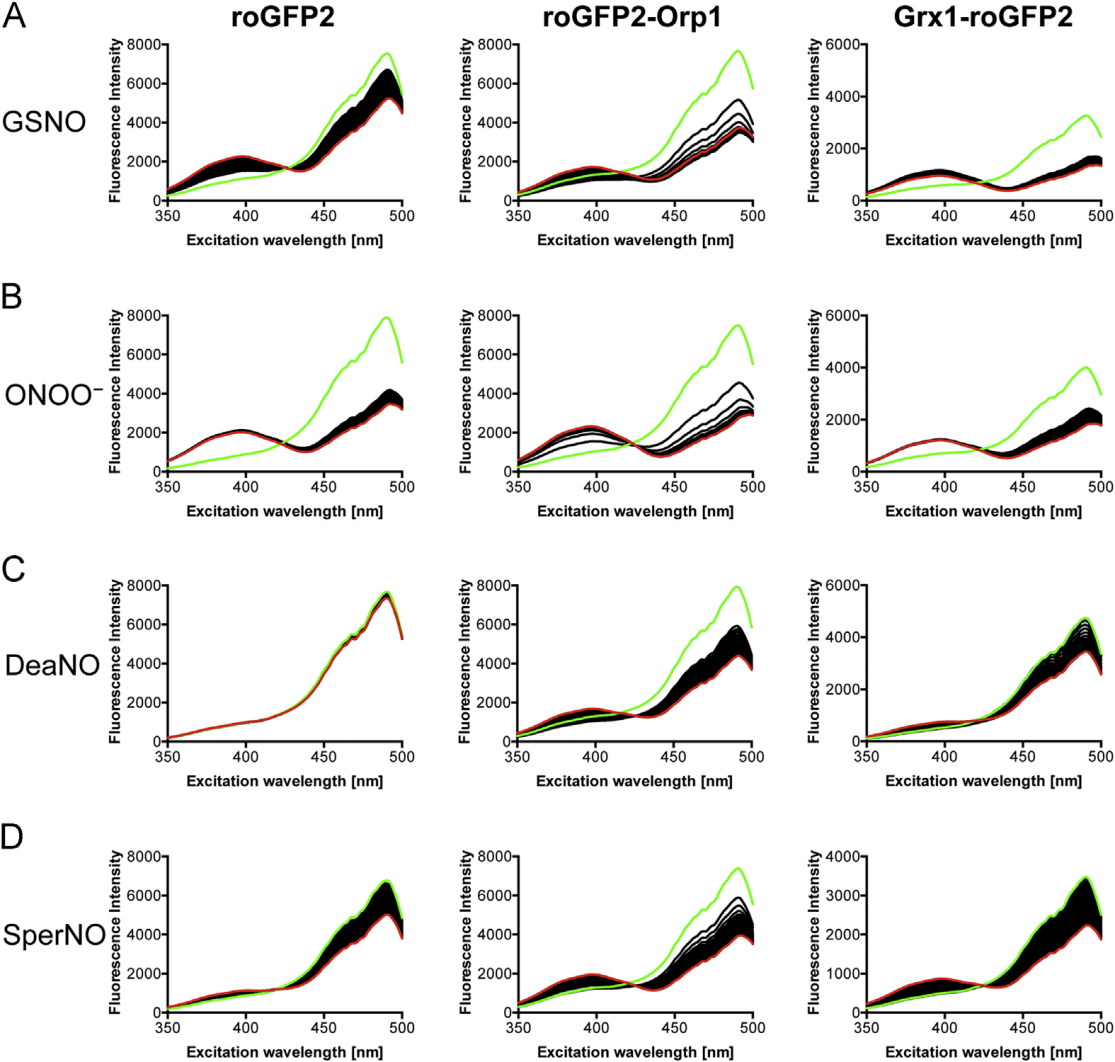
Response of the three probes to various nitrosative agents. The indicated compounds were added at a final concentration of 100 µM after measurement of the first spectrum (green). Subsequently, a total of 60 spectra were recorded. The last recorded spectrum is shown in red.

**Fig. 10:**
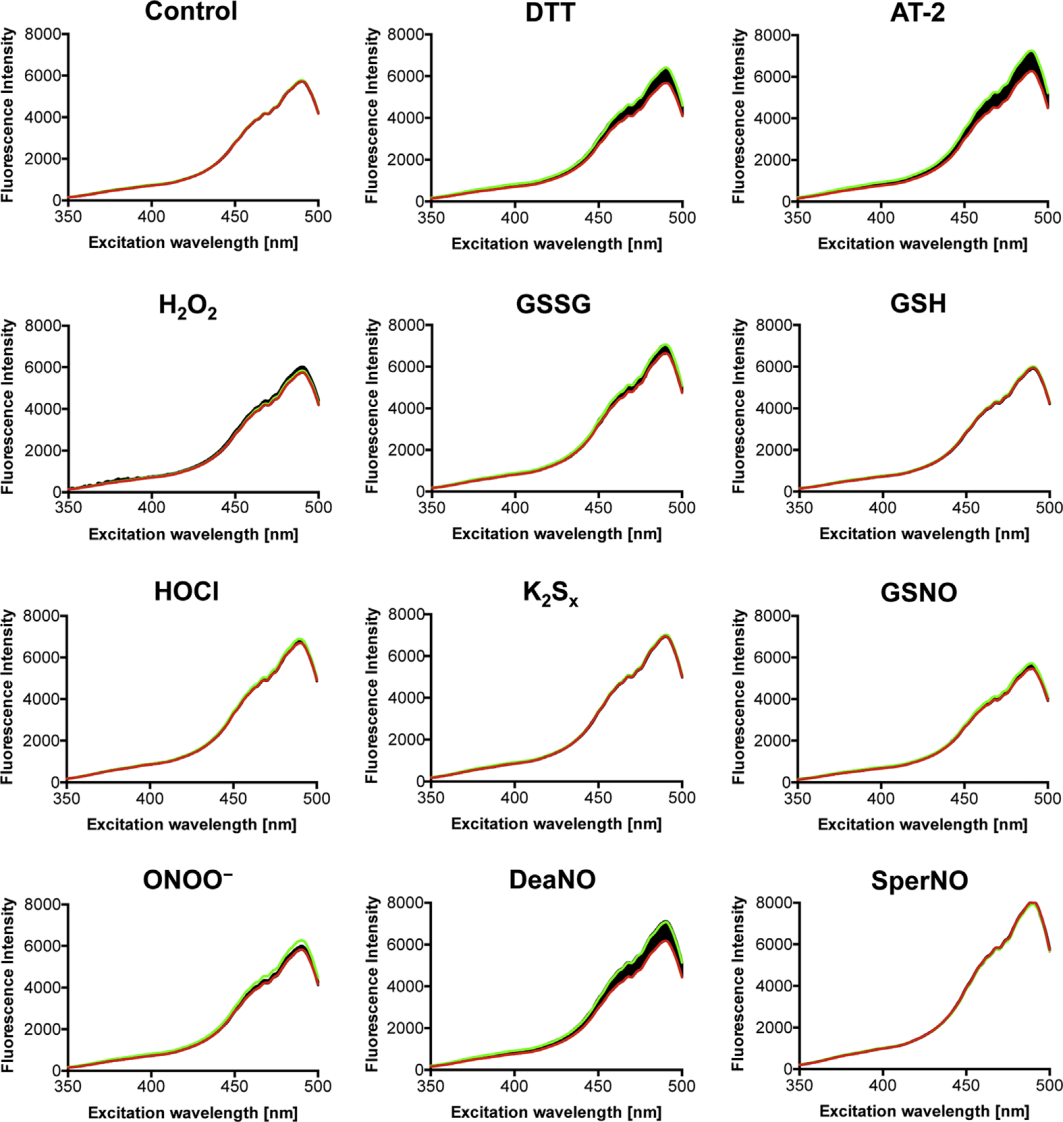
Response of eGFP to the compounds used in this study. The response of eGFP to the compounds used in this study was measured to rule out general effects of these compounds on GFP fluorescence characteristics. eGFP was added to PBS to a final concentration of 0.2 µM. After the measurement of one spectrum (green), 2 µM of the compounds were added and a total of 60 spectra were recorded. The last recorded spectrum is shown in red.

Each experiment represents a time series of 60 fluorescence excitation spectra with one spectrum recorded per minute. The first spectrum shows the fluorescence excitation spectrum prior to treatment. Following the addition of oxidative or nitrosative species, spectral changes of roGFP2, roGFP2-Orp1, and Grx1-roGFP2 were recorded for a total of 60 min. Autoxidation of roGFP2-Orp1 under aerobic conditions is shown in Fig. 1. Experiments involving roGFP2-Orp1 were thus performed under anaerobic conditions, while measurements with roGFP2 and Grx1-roGFP2 were performed under aerobic conditions. Reference spectra were recorded using 2 µM of control reductant (DTT or GSH), or 2 µM of control oxidant (AT-2) (Fig. 2). The specificity of the two fusion probes roGFP2-Orp1 and Grx1-roGFP2 was tested using 2 µM H_2_O_2_ and GSSG (Fig. 3). Spectral responses to 2 µM of various other oxidative and nitrosative species were recorded (Fig. 4). Unfused roGFP2 was treated with increasing concentrations of H_2_O_2_, HOCl, and ONOO^−^ to determine the minimal oxidant concentration eliciting a spectral response (Fig. 5, Fig. 6, Fig. 7). The response of the three probes to treatment with 100 µM of the above tested oxidants was recorded (Fig. 8, Fig. 9). To rule out artifacts, non-redox-sensitive eGFP was treated with all compounds used in this study (Fig. 10). The raw data for all figures (excitation wavelength vs. fluorescence intensity values) is made available as comma separated value (.csv) text files.

## 2 Experimental design, materials and methods

Purification and reduction of roGFP2, roGFP2-Orp1, and Grx1-roGFP2, and preparation of oxidants is described in [1]. Purification of eGFP, heterologously expressed in *E. coli* BL21 from plasmid pET16b_eGFP SC+ [2] was essentially performed as described for the roGFP2-base probes [1]. roGFP2 or Grx1-roGFP2 was added to a 1,500 µL QS fluorescence cuvette with a stirring bar containing 1 mL of buffer (PBS, 5 mM EDTA, pH 7.4) at a final concentration of 0.2 µM. Measurements of roGFP2-Orp1 were performed under anaerobic conditions as described in [1]. Measurements were done at 20 °C in a JASCO FP-8500 fluorescence spectrometer equipped with a Peltier thermo-holder ‘EHC-813’ at 20 °C for 60 min under continuous stirring. Measurement parameters were as follows: 510 nm (Em), 350–500 nm (Ex), 5 nm slit width (Ex/Em), medium sensitivity. After a single recorded spectrum, oxidants were added at the indicated final concentrations and a time series of 59 additional spectra was recorded. Expression of roGFP2-Orp1 in *E. coli* MG1655 (Fig. 1F) was performed as described in [1].
